# Detecting Bone Marrow Edema of the Extremities on Spectral Computed Tomography Using a Three-Material Decomposition

**DOI:** 10.3390/diagnostics13172745

**Published:** 2023-08-24

**Authors:** Marie Schierenbeck, Martin Grözinger, Benjamin Reichardt, Olav Jansen, Hans-Ulrich Kauczor, Graeme M. Campbell, Sam Sedaghat

**Affiliations:** 1 Department for Radiology and Neuroradiology, University Hospital Schleswig-Holstein Campus Kiel, 24105 Kiel, Germany; 2 German Cancer Research Center, University Hospital Heidelberg, 69120 Heidelberg, Germany; 3 Department of Interventional Radiology and Neuroradiology, Klinikum Hochsauerland, 59821 Arnsberg, Germany; 4 Department of Diagnostic and Interventional Radiology, University Hospital Heidelberg, 69120 Heidelberg, Germany; 5 Clinical Science, Philips GmbH Market DACH, 22335 Hamburg, Germany

**Keywords:** bone marrow edema, fracture, computed tomography, trauma, extremity

## Abstract

Background: Detecting bone marrow edema (BME) as a sign of acute fractures is challenging on conventional computed tomography (CT). This study evaluated the diagnostic performance of a three-material decomposition (TMD) approach for detecting traumatic BME of the extremities on spectral computed tomography (SCT). Methods: This retrospective diagnostic study included 81 bone compartments with and 80 without BME. A TMD application to visualize BME was developed in collaboration with Philips Healthcare. The following bone compartments were included: distal radius, proximal femur, proximal tibia, distal tibia and fibula, and long bone diaphysis. Two blinded radiologists reviewed each case independently in random order for the presence or absence of BME. Results: The interrater reliability was 0.84 (*p* < 0.001). The different bone compartments showed sensitivities of 86.7% to 93.8%, specificities of 84.2% to 94.1%, positive predictive values of 82.4% to 94.7%, negative predictive values of 87.5% to 93.3%, and area under the curve (AUC) values of 85.7% to 93.1%. The distal radius showed the highest sensitivity and the proximal femur showed the lowest sensitivity, while the proximal femur presented the highest specificity and the distal tibia presented the lowest specificity. Conclusions: Our TMD approach provides high diagnostic performance for detecting BME of the extremities. Therefore, this approach could be used routinely in the emergency setting.

## 1. Introduction

Bone fractures are common conditions that present in the routine emergency setting. The accurate diagnosis of fractures after acute trauma is essential for adequate consecutive treatment. This can be challenging in the case of detecting occult fractures. Acute trauma can cause microfractures in the trabecular bone, leading to bone marrow edema (BME) and microhemorrhages, eventually causing a reduction in fat components [1]. Although BME is not specific to fractures, the presence of BME in acutely injured bone indicates the presence of a fracture [2,3]. To appreciate the importance of BME as an indirect sign of a fracture, Baumbach et al. developed a workflow for BME management and decision-making, placing the presence of BME at the beginning of their decision algorithm [3]. The changes in the fluid content of the bones causing BME are best diagnosed with magnetic resonance imaging (MRI), which reveals reduced signal intensity on T1-weighted (w) images and increased signal intensity on fat-suppressed T2w or proton density-weighted (PDw) images [4,5]. MRI is often performed to identify BME, which many authors consider highly specific for acute trauma [6,7]. Therefore, it is not surprising that MRI has become a standard imaging modality for assessing bone marrow edema in the emergency setting [5]. However, contraindications, such as implemented medical devices, ferromagnetic materials, claustrophobia, or severe back pain, often restrict the use of MRI [8,9]. Additionally, MRI examinations are time-consuming and usually not immediately available in an emergency setting [10]. Patients with acute trauma of the extremities often require immediate operative treatment. Therefore, computed tomography (CT) is often the imaging modality of choice in emergency situations, as the examination times are faster, hence the susceptibility to patients’ motion is reduced [8,11]. With traditional CT scanners, which are based on a single-energy X-ray tube and detector, the diagnosis of BME is often very challenging, as for low-contrast tissues like bone marrow, the discrimination from normal bone is aggravated by a superposition of the X-rays and the overlying trabecular bone [12,13,14,15]. An advanced technique using dual-energy computed tomography (DECT) has been employed for many years to provide additional information on different tissue characteristics. Different approaches for DECT, such as rapid kilovoltage (kV) switching or two X-ray sources, have already been described in the literature [7]. The kV-switching method uses a single X-ray tube with the ability to rapidly change the energy levels [10]. A major disadvantage of using a DECT system was previously seen in higher radiation doses [16]. However, further developments have led to significantly reduced radiation doses [17]. Apart from these source-based systems, there is also a detector-based approach for material separation by their energy-dependent X-ray absorption characteristics. A novel technique called dual-layer spectral CT (SCT), using one X-ray tube and two different detector layers, was introduced recently, which registers different energy spectra of the polychromatic X-ray spectrum [7,18,19]. The top layer absorbs low-energy spectra and the bottom layer absorbs high-energy spectra [12]. This allows for the simultaneous absorption of the polychromatic X-ray beam’s high- and low-energy spectra. In this way, the discrimination of tissues by different atomic weights and electron densities is acquired [7,13,18,19]. Various post-processing approaches and other image reconstruction methods using SCT have already been described in the literature [7,13,18,19]. Post-processing SCT image reconstructions allow for the visualization of different tissues, leading to selective imaging of tissue changes in different clinical scenarios. BME using SCT and DECT has already been investigated in several studies [4,11,14,20,21]. Also, BME of the extremities has been described in previous studies using DECT [22,23], and one of these studies also used a three-material decomposition (TMD) approach [22]. However, no study has addressed the detection of BME of the extremities using SCT. Therefore, the applicability of TMD on SCT to detect BME in extremity bones is an interesting and relatively novel issue to investigate. In summary, this study evaluated the diagnostic performance of a TMD approach to visualize BME of the extremities using SCT.

## 2. Materials and Methods

Study design and IRB approval: This is a retrospective monocentric study. The study was approved by the local institutional review board (IRB) of our institution. Informed consent was waived due to the retrospective design of the study and the inclusion of patients who had undergone their examinations during routine clinical examinations. The study was conducted between 2019 and 2022.

**Figure 1:**
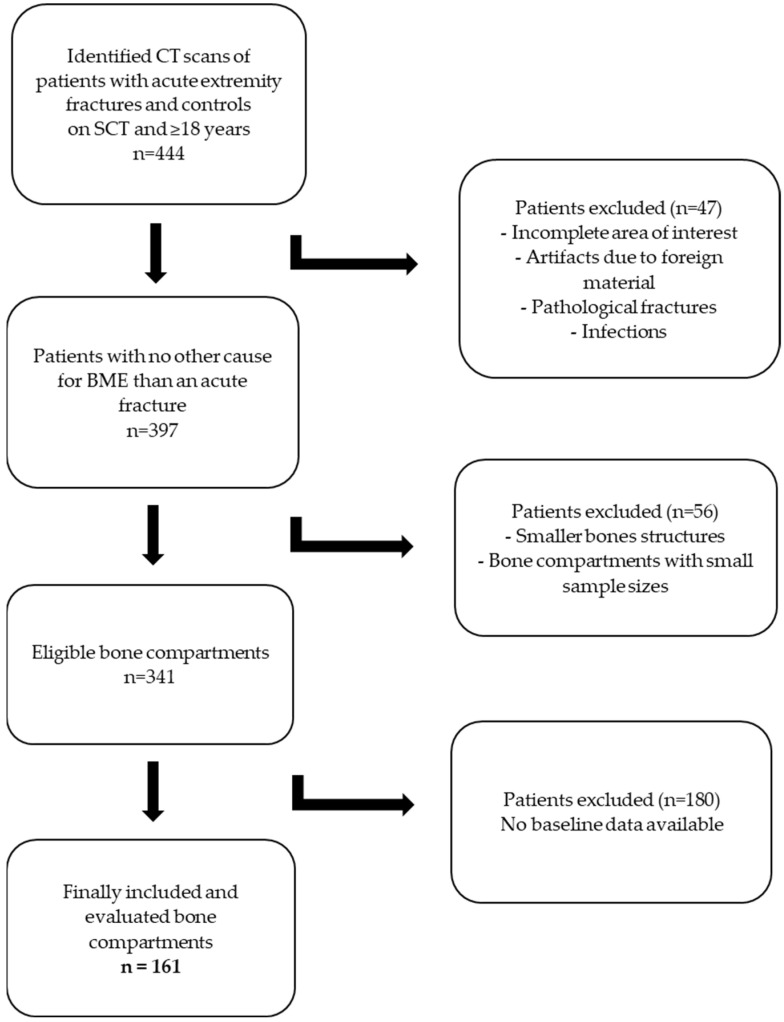
Flowchart of the study selection process based on the inclusion and exclusion criteria.

Patients: Our institutional picture archiving and communication system (PACS) was screened for patients who underwent SCT of the extremities within the study period. First, the patients who presented with extremity fractures were identified. In the second step, control patients without any fractures of the extremities were included. All available spectral-based images (SBI) from the patients were downloaded and reconstructed for the subsequent BME analysis (as indicated under the subheading SCT and BME imaging). Before the main evaluation took place, we performed a two-step pre-evaluation. First, we screened all fracture sites for the number of occurrences. From this list, we excluded all fracture sites with a low number (n). In the second step, we reconstructed all available SBI datasets from the remaining patients and pre-evaluated the performance of our TMD approach. Our pre-evaluation of the TMD tool showed that small edemas, as well as smaller bone structures (carpal or tarsal bones), could not be reliably identified using our TMD approach. Therefore, small edemas and small bone structures were excluded. Patients who underwent CT examinations other than SCT (e.g., dual-energy CT) were excluded as well. Also, patients who presented with foreign materials causing artifacts, those aged under 18 years, those with pathological fractures, and those with infections of the extremities were excluded. We performed a pre-selection of fractures based on the most common kinds of fractures scanned with SCT. According to our pre-selection, the following body compartments remained for evaluation: distal radius, proximal femur, proximal and distal tibia, and long bone diaphysis (humerus, femur, tibia, radius). As patients’ baseline data, age and sex were included. All other patient-related data were not evaluated. Figure 1 shows a flowchart of the study selection process based on the inclusion and exclusion criteria.

SCT and BME imaging: The patients were examined using dual-layer spectral computed tomography (Spectral IQON^®^, Philips Healthcare, Best, The Netherlands) without i.v. contrast application. CT images were acquired using a tube voltage of 120 kV, an automated attenuation-based dose modulation (DoseRight, Philips Healthcare), a rotation time of 0.27 s, a collimation of 64 × 0.625, and a slice thickness of 1 mm. Spectral-based image (SBI) data, which contain information on energy-dependent absorption, were reconstructed. For further image reconstructions, a post-processing platform (IntelliSpace, Philips Healthcare) was used. A plug-in was implemented to generate the evaluated TMD tool using the following process: The dual-layer attenuation data were decomposed into photo-electric (PE) and Compton-scatter (CS) attenuation, which provides the basis for the TMD method. PE and CS values for three materials (water, fat, and bone mineral) in pure form were set as inputs to the algorithm, after which the volume fraction of each material in each voxel was calculated, knowing the total PE and CS attenuation of that voxel. This resulted in a BME tool, where the voxel values represent the water volume fraction in each voxel. This method assumes that the sum of the volume fractions of each material is equal to one. TMD images were reconstructed using iDose level 2 (Philips Healthcare, The Netherlands) and Spectral level 2 (Philips Healthcare, The Netherlands). The density maps for the water volume fractions were generated in different orientations (according to the original CT image) and with a section thickness of 3 mm. The reconstruction resulted in grayscale images, simulating the contrast of MR images.

Image Analysis: Datasets of bones with and without BME were reconstructed and prepared by M.S. for consecutive review and analysis. The images were downloaded from our institutional PACS and saved in DICOM (digital imaging and communications in medicine) files. Two radiologists (readers) with 5 and 15 years of experience in musculoskeletal radiology evaluated the prepared datasets. The radiologists were blinded to the data and reviewed the TMD images independently in a random order. They indicated the presence of BME using a binary classification (0 no edema visible, 1 edema visible). Only clearly visible BME had to be rated as “1” according to the binary rating. The radiologists were allowed to scroll through the reformations and adjust the window for personal convenience. The cases were randomly chosen. MRI was used as a baseline to determine the presence or absence of BME.

Statistical analysis: Data were provided as mean values with standard deviation (SD). Interrater reliability was calculated with weighted Cohen’s κ statistics. The diagnostic performance (sensitivity, specificity, positive predictive value, and negative predictive value) was assessed using contingency tables and then calculated. The area under the curve (AUC) was also derived from the receiver operating characteristic (ROC) curve analysis. From the BME ratings of the readers, mean values between the ratings were calculated and used for the determination of the diagnostic performance. Statistical significance for all tests was set at *p* < 0.05. Statistical analysis was performed using the IBM-SPSS version 28.0 software package (IBM, Armonk, NY, USA).

## 3. Results

### 3.1. Baseline Data

This study included 161 bone compartments—81 bone compartments with and 80 bone compartments without BME. The fracture locations are shown in Table 1. The locations presented with total numbers between *n* = 29 and *n* = 35. The mean age of the patients was 59.5 years (SD 21.9); 51% of the patients were male and 49% were female. The inter-rater reliability was 0.84 (*p* < 0.001).

True/false positive/negative results using the TMD approach

Altogether, 73 of the 81 BME were correctly identified. Only 16 cases were falsely classified as positive or negative (*n* = 8 each). Out of the 80 bones without BME, 72 were correctly classified. Altogether, 90% of the bone compartments were correctly classified by the two readers.

Calculated diagnostic performance using the TMD approach

Table 2 presents the diagnostic performance values of the TMD tool.

The different bone compartments showed sensitivities of 86.7% to 93.8%, specificities of 84.2% to 94.1%, positive predictive values of 82.4% to 94.7%, negative predictive values of 87.5% to 93.3%, and area under the curve (AUC) values of 85.7% to 93.1%.

The distal radius showed the highest sensitivity (93.8%) and the proximal femur showed the lowest sensitivity (86.7%), while the proximal femur presented the highest specificity (94.1%) and the distal tibia presented the lowest specificity (84.2%). The highest AUC was seen in the long bone diaphysis (93.1%), while the distal tibia showed the lowest AUC (85.7%). There was no diagnostic value lower than 80%.

### 3.2. Examples

**Figure 2:**
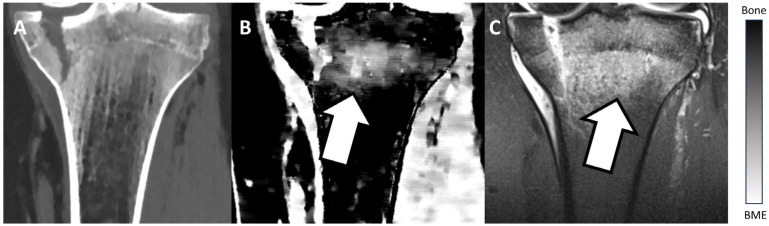
Fractured proximal tibia of a patient on a conventional CT image (**A**), on the TMD tool (**B**), and the corresponding MRI (**C**). The BME (white arrow) is visible on the TMD tool and the corresponding MRI, but it is not detectable on the conventional CT image. Additionally, a bar chart shows the density/intensity levels between normal bone and BME.

**Figure 3:**
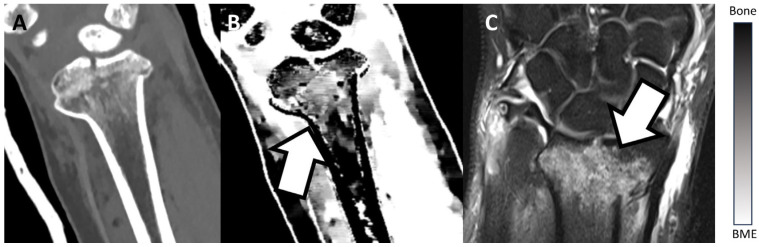
Fractured distal radius of a patient on a conventional CT image (**A**), on the TMD tool (**B**), and the corresponding MRI (**C**). The TMD tool and the corresponding MRI clearly show a BME (white arrow), whereas the conventional CT image does not depict the BME. Additionally, a bar chart shows the density/intensity levels between normal bone and BME.

Figure 2 and Figure 3 show examples of BME on TMD images in different locations. Figure 2 presents an example of a fractured proximal tibia on conventional CT images and the TMD tool, whereas Figure 3 shows a fractured distal radius.

**Table 2:** Diagnostic performance showing sensitivity, specificity, positive predictive value (PPV), negative predictive value (NPV), and area under the curve (AUC) (all in %) (combined for both readers).

	Sensitivity	Specificity	PPV	NPV	AUC
Distal radius	93.8%	85.7%	88.2%	92.3%	90.0%
Proximal femur	86.7%	94.1%	92.9%	88.9%	90.6%
Proximal tibia	90.0%	93.3%	94.7%	87.5%	91.4%
Distal tibia	87.5%	84.2%	82.4%	88.9%	85.7%
Long bone diaphysis	92.9%	93.3%	92.9%	93.3%	93.1%

## 4. Discussion

This study investigated the diagnostic performance of a TMD approach for detecting BME of the extremities on SCT. Although extremity fractures are commonly seen after trauma, there is still a lack of studies systematically addressing extremity fractures [24]. Additionally, many extremity fracture studies are outdated or include only a few patient samples [25]. Most upper extremity fractures in older patients are caused by a fall and a subsequent direct fracture impact [26]. Also, many other fracture mechanisms exist, such as stress fractures [27,28].

BME plays an essential role in diagnosing acute fractures [29,30]. Although BME is not a specific characteristic of acute fractures and it might appear in both symptomatic and asymptomatic individuals [2], BME is routinely seen in acute traumatic bone. Therefore, BME is often regarded as an indirect sign of acute fractures [3,29,30].

Radiological diagnostics is one of the major pillars for identifying traumatic fractures. Radiography is usually performed first, followed by CT. Computed tomography is nowadays established as an imaging modality that provides reliable fracture diagnostics [31,32]. However, the utilization of conventional CT is unrewarding for detecting BME, as superposition aggravates the discrimination of low-contrast tissues [12,13]. This is a well-known problem for radiologists and other clinical disciplines. However, many radiologists try to provide some vague information about fracture age, mainly based on their personal experience [33]. This could cause radiologists to be accused of misdiagnosis or failure to diagnose [34], although they only wanted to help their patients. Consequently, radiologists should try to reduce errors as much as possible. Reducing errors will improve patient care and reduce healthcare costs [34]. Therefore, in routine clinical settings, MRI is often used in undefined cases to detect BME. Nevertheless, MRI examinations are often not immediately available in the emergency setting and additionally, many patients are ineligible for MRI [7,10]. Although only a few fractures are missed in the emergency setting [35], occult fractures still cause a major problem for radiologists. Occult fractures are often difficult to detect on CT, leading to misdiagnosis or further diagnostics. Therefore, in an emergency, where time saves lives, it is preferable to detect occult fractures, indicated by BME, on-site without needing additional MRI [36,37].

Depending on the hardware setup, there are different ways to generate spectral information based on dual-energy CT [7]. Dual-energy with dual-source CT, using either different X-ray sources or rapid kV switching [7], has been available and clinically tested for many years [10,38]. The SCT consists of two detector layers, which simultaneously collect low-energy and high-energy data, consecutively allowing for the separation of these different energy spectra [1,13,18,19]. This relatively novel technique makes multiple reconstructions of various structures within the human body possible. One of the many advantages of SCT compared to DECT is that SCT allows for the analysis of spectral information without requiring specific predefined protocols [7]. The idea of a three-material decomposition for BME visualization is not new, and a similar approach has already been investigated for DECT [22]. Accordingly, Yadav et al. assessed the diagnostic accuracy of DECT in detecting bone marrow edema in patients with trauma of the lower limb. They included both virtual non-contrast (VNC) and TMD approaches, and eventually found sensitivities and specificities of 94.1% and 91.3%, respectively [22]. However, there is no previous study that evaluates a TMD approach for use in the extremity bones on SCT. Our TMD approach was developed in collaboration with the manufacturer. Also, “self-made” approaches using the same technique are possible, especially when there is no SCT available. Post-processing tools, such as our TMD approach, enhance the quality of imaging and allow for more accurate diagnostics of targeted tissues. The potential of many SCT reconstructions and applications has yet to be fully utilized. Previous studies on BME detection in SCT mainly focused on post-processing imaging using CaSupp, which relies primarily on suppressing calcium as a central component of bones and consecutively making BME visible [4,13,14,39]. In contrast to this, our approach allows for the direct visualization of BME on SCT, compared to CaSupp. Our TMD method enables BME depiction by evaluating each voxel’s water volume fraction. Additionally, previous SCT studies mainly focused on vertebral fractures rather than fractures outside the vertebrae [7,11,13,21,37,39]. However, fractures of the extremities, with their various locations, are among the most common fracture sites [40,41].

In this study, we achieved high diagnostic performance of the TMD tool in identifying BME in major bone structures of the extremities, subsequently allowing for an accurate evaluation of the fracture acuity. Despite the high diagnostic performance of our TMD tool, the interrater reliability was also high, allowing for the reliable and objective detection of BME. Nevertheless, we performed a pre-selection and pre-evaluation of the included fractures. The pre-evaluation revealed that our TMD approach could only be used for major bone compartments of the extremities. Small bone structures and small edemas can be hard to identify using our approach.

Taking our results into account, our TMD approach could be used in the routine emergency setting for major bone compartments of the extremities and acute fractures in these compartments. Consequently, patients with clinically acute extremity fractures could be identified earlier and more precisely compared to conventional CT, in which BME is typically not visible. This could be very helpful, especially in occult fractures. Also, time-consuming MRI for fracture evaluation could recede into the background, eventually leading to a more time-effective treatment of trauma patients. Baumbach et al. presented an algorithm and decision tree for BME evaluation [3]. Acute fractures thereby presented a main point of their algorithm, with BME among the first appearances and features to be reviewed. The first step was described to be the pain, followed by an MRI, which shows the BME as an indirect sign of an acute fracture. Thereafter, comes CT or X-ray. By using our TMD approach, CT could rank more importantly in the algorithm as a first-line diagnostic tool. However, our TMD tool cannot fully replace MRI, and our approach is still a concept/prototype at the time of this study. Further development and exciting features, such as color-mapping or quantitative voxel-based analysis, could improve the performance of our tool. Future studies should test our approach by including many more patients. Also, automated post-processing TMD reconstruction after trauma CT, without any further manual reconstruction, could be an interesting aim for future studies.

This study has some limitations. First, we performed a retrospective study. Future studies could perform the same study in a prospective design and include many more patients. Second, we excluded other BME-causing conditions, such as metabolic diseases or malignancies. However, in the emergency setting, these types of patients could still be encountered. Therefore, future studies could focus on the evaluation of trauma-related and non-trauma-related BME. Third, many eligible patients did not undergo MRI baseline examinations, and they had to be excluded from this study. In fact, MRI examinations are often not performed in an emergency setting to avoid treatment delays, leading to a lack of baseline MRI data. Fourth, we did not include smaller bone structures, such as carpal and tarsal bones, as a pre-evaluation of our tool revealed difficulties in depicting BME in those smaller bone compartments. Future studies should use our TMD approach and optimize it for smaller bone structures. Lastly, the developed and evaluated TMD tool is still a concept or prototype. For future developments, an easy-to-handle interface [42] or an automated approach could be very helpful in making our TMD approach broadly usable in clinics.

## 5. Conclusions

We developed and tested a novel TMD approach for making BME of the extremities more visible on SCT. Our TMD approach revealed high diagnostic accuracy in detecting BME of the extremities, with sensitivities and specificities ranging from 86.7% to 93.8% and 84.2% to 94.1%, respectively. Our approach could facilitate diagnostics of acute extremity fractures, especially in occult fractures, consecutively leading to a faster onset of therapy. However, the presented TMD tool is not suitable for small bone structures, as BME is usually not visible in those bone compartments. Further studies could focus on testing the proposed TMD tool in larger cohorts or routine clinical settings and could include additional body compartments not evaluated in this study.

## Figures and Tables

**Table 1:** Overview of cases with and without bone marrow edema (BME) (*n* = number).

Location	BME (*n*)	No BME (*n*)	Total (*n*)
Distal radius	16	14	30
Proximal femur	15	17	32
Proximal tibia	20	15	35
Distal tibia	16	19	35
Long bone diaphysis	14	15	29
Total	81	80	161

## Data Availability

Data cannot be provided due to ethical reasons.
